# MACC-1 Promotes Endothelium-Dependent Angiogenesis in Gastric Cancer by Activating TWIST1/VEGF-A Signal Pathway

**DOI:** 10.1371/journal.pone.0157137

**Published:** 2016-06-09

**Authors:** Lin Wang, Rui Zhou, Yang Zhao, Shaoting Dong, Jingwen Zhang, Yuhao Luo, Na Huang, Min Shi, Jianping Bin, Yulin Liao, Wangjun Liao

**Affiliations:** 1 Department of Oncology, Nanfang Hospital, Southern Medical University, Guangzhou, China; 2 Department of Cardiology, Nanfang Hospital, Southern Medical University, Guangzhou, China; Cincinnati Children's Hospital Medical Center, UNITED STATES

## Abstract

Endothelium-dependent angiogenesis is thought to be a crucial step in cancer progression. We previously reported that metastasis-associated in colon cancer-1 (MACC1) contributed to the vasculogenic mimicry in gastric cancer (GC), but it remains unknown whether MACC1 promotes endothelium-dependent angiogenesis of GC and whether TWIST1 is involved in this process. In the present study, we detected MACC1 expression and microvessel density (MVD) by immunohistochemistry in 159 patients with stage I-III GC, and investigated the role of TWIST1 and vascular endothelial growth factor A (VEGF-A) in MACC1-induced endothelium-dependent angiogenesis using nude mice with GC xenografts, and human umbilical vein endothelial cells (HUVECs) that were co-cultured with conditioned media from overexpression and interference MACC1 GC cells. We found that MACC1 expression was positively correlated with an increased MVD and tumor recurrence in GC patients. In GC xenograft models, MACC1 elevated MVD and upregulated the expression of VEGF-A as well as accelerated tumor growth. In addition, MACC1 obviously increased the expression of TWIST1 and induced tube-like formation of HUVECs, whereas attenuation of TWIST1 suppressed the protein expression of VEGF-A and repealed the effect of MACC1 on tube formation. Our findings shed light on the function of MACC1 in endothelium-dependent angiogenesis of GC and suggest potential prognostic and therapeutic value.

## Introduction

Continuous blood supply is necessary for the rapid growth of tumors[1]. Besides the well-studied endothelium-dependent angiogenesis, recent studies have revealed several new patterns including vasculogenic mimicry and mosaic vessels[2, 3]. We previously reported that metastasis-associated in colon cancer-1 (MACC1) contributed to the vasculogenic mimicry (VM) formation in human gastric cancer (GC)[4]. However, it remains unknown whether MACC1 is involved in the true endothelium-dependent angiogenesis of GC.

MACC1, as a key regulator of hepatocyte growth factor (HGF)/c-Met/mitogen-activated protein kinases (MAPK) signaling pathway[5–7], promotes the proliferation, migration, and invasion of GC cells[8], as well as affecting the tumor glycometabolism[9] and microenvironment[4, 10, 11], at last predicting poor clinical outcome for GC patients[8]. Previous studies reported that multiple regulators of VM[12, 13] participated in angiogenesis[14, 15]. Therefore, we highly speculate that MACC1 plays an important role in the endothelium-dependent angiogenesis of GC.

It is reported that angiogenesis is closely related with post-surgery tumor recurrence[16]. But it is not clear whether combination of MACC1 and high density of microvessel (MVD) further influence disease-free survival (DFS) of GC patients, nor the molecular mechanism of MACC1-induced angiogenesis. In our preliminary study, we showed that TWIST1 was necessary for MACC1-induced VM in GC[4], while TWIST1 expression promoted vascularization of breast carcinoma[17, 18] and supported the vascular development by upregulating vascular endothelial growth factor (VEGF)-A[19], which is the first form of identified pro-angiogenic factor[20, 21]. These results strongly suggest that TWIST1/VEGF-A angiogenic axis has a central role in endothelium-dependent angiogenesis of tumors. Importantly, previous reports about the analysis of published microarray datasets showed that MACC1 mRNA levels were significantly correlated with VEGF-A expression in GC tissues (TCGA; n = 387, r = 0.224, P < 0.001) (S1 Fig). Above all, we hypothesize that MACC1 promotes endothelium-dependent angiogenesis via activating TWIST1/VEGF-A signaling axis in GC.

In this study, we examined whether MACC1 was positively associated with endothelium-dependent angiogenesis. We detected whether MACC1-positive expression and high MVD predicted short DFS in patients with GC after radical resection. Then, we investigated the role of MACC1 in angiogenesis in vivo and in vitro. Finally, we determined whether TWIST1/VEGF-A angiogenic axis represented the underlying mechanism of MACC1-induced endothelium-dependent angiogenesis.

## Materials and Methods

### Patients and Tumor Tissue Samples

Paraffin-embedded pathological specimens were obtained from 159 patients with Stage I-III GC that underwent radical surgical resection at Nanfang Hospital of Southern Medical University (Guangzhou, Guangdong, China) between 2005 and 2010. All the patients were diagnosed according to the 7 th edition proposed by American Joint Committee on Cancer Manual (AJCC) 2010 staging system of GC. We confirmed that all experiments were performed in accordance with the relevant guidelines and informed consent was obtained from all subjects. The study protocols were approved by the Ethical Committees at Nanfang Hospital and written informed consent was obtained from each patient.

### Animal Models

Male NOD-SCID nude mice of 5-week-old were obtained from Sun Yat-Sen University (Guangzhou, China). Stable overexpression of MACC1 gene (oxMACC1) and vector-control cells or silencing of MACC1 (shMACC1) and scramble-control GC cells (1 × 10^7^ cells/mouse, n = 6) were injected subcutaneously into the left and right sides of the back as previously described[8]. Tumor nodules were monitored every 3 days by caliper measurements of the length and width of the tumors. Tumor volumes were calculated according to the formula: Volume = width × length × (width + length)/2. All mice were housed in a specific pathogen-free facility in microisolator cages with free access to autoclaved food and acidified water supplemented with sulfamethoxazole–trimethoprim. All animal experiments were approved by the Laboratory Animal Administration Committee of Nanfang Hospital (Permit Number: NFYY-2015-10) and consistent with the Guide for the Care and Use of Laboratory Animals published by the US National Institutes of Health. Tumors were allowed to grow to 0.5–1 cm in size in the largest dimension and were harvested on day 18, because huge tumor volume was prone to central necrosis of tumors that affected the results in the following experiments[8]. Then, tissue collection procedures were initiated after animals had been euthanized by cervical dislocation. Mice were removed from their cages and gently restrained while resting on the benchtop. Cervical dislocation was performed manually and resulted in euthanasia within approximately 10 seconds. The subcutaneous tumors were used for immunohistochemical (IHC) staining.

### Human Vascular Endothelial Cell Tube Formation Assay

HUVECs tube formation assay was performed by pipetting 200 μl Matrigel (BD Biosciences, Franklin Lakes, NJ, USA) into each well of 24-well plate, which was then polymerized for 1 h at 37°C. HUVECs (1 × 10^5^) with 200 μl conditioned medium were added into each well and incubated for 12 h. Images were taken using a bright-field microscope at a magnification of 100 ×. The capillary tubes were quantified by counting the average numbers of completed tubule structures in three randomly selected fields.

### Cell Lines and Cell Cultures

OxMACC1 and shMACC1 gastric adenocarcinoma cell lines were carried out as described in our previous study[8]. Cells were maintained with Dulbecco’s modified Eagle medium (Invitrogen, Carlsbad, CA, USA) containing 10% fetal bovine serum (FBS) (HyClone, Logan, UT, USA), and 0.5 μg/mL puromycin[8]. Cell lines were authenticated by short tandem repeat and genotyped upon re-expansions, and experiments were carried out with low passage cultures of these stocks. HUVECs were obtained from Yiyuan Biotechnology Company (Guangzhou, China), and cultured in endothelial cell growth media (Gibco, Carlsbad, CA, USA) supplemented with 0.1 mg/ml heparin and 20% FBS (HyClone) according to the supplier’s suggestions. For experiments, cells were used between 2 and 5 passages.

### Plasmids and Cell Transfection

Human TWIST1 was amplified by PCR and cloned into the pcDNA3.1 vector (Primer sequences in S1 Table) (Invitrogen), and the recombinant plasmid pcDNA3.1/TWIST1 was constructed. The silencing efficiency of three pairs TWIST1-specific siRNA(Sigma, St. Louis, MO, USA) in ORF region was examined by quantitative real-time PCR (qRT-PCR, Primer sequences in S2 Table). GC cells were transfected with pcDNA3.1/Twist1 and TWIST1-specific siRNA using Lipofectamine 2000 (Invitrogen).

### Immunohistochemistry Staining

IHC staining was performed according to the standard protocol[8]. The sections were incubated with a series of primary and secondary antibodies (S3 Table). Staining scores were evaluated by three independent reviewers, and the half-quantitative scoring system was determined as reported elsewhere[8]. Briefly, for MACC1 staining intensity, sections were scored as 0 (negative), 1 (weak), 2 (medium), or 3 (intense). While the staining extent was scored according to the area percentages: 0 (0%), 1 (1%–25%), 2 (26%–50%), 3 (51%–75%), or 4 (76%–100%). The products of the staining intensity and extent scores were the final staining scores (0–12) for MACC1 expression. Tumors of final staining score ≥ 3 were considered to be positive expression because 95% of normal gastric tissues expressed low level of MACC1 with an IHC score of < 3 in our preliminary study[8]. We further defined 3 ~ 7 as low expression and 8 ~ 12 as high expression in order to perform qualitative analysis.

### Microvessel Density

MVD was determined as described by Weidner et al.[22]. MVD was assessed according to platelet endothelial cell adhesion molecule-1 (CD31) IHC staining of tumor vessels. The sections were initially scanned at low power objective (magnification 100 ×) to select the most vascularized (hot-spots) areas. The microvessels in the hot-spots were then counted at a magnification of 200 ×, and the average vessel count in six hot-spots was calculated as the MVD. All counts were independently reviewed by three observers who were blinded to the corresponding clinicopathologic data. The MVD was classified as either high (≥ 36.8) or low (< 36.8); 36.8 was the median value of MVD.

### Enzyme-Linked Immunosorbent Assay

The concentration of TWIST1 and VEGF-A were quantitated using a commercially available TWIST1 (Sandwich, Lifespan Biosciences, CA, USA) and VEGF-A (R&D Systems, Minneapolis, MN, USA) enzyme-linked immunosorbent (ELISA) kit according to the manufacturer’s instructions. The results presented the mean values from three separate experiments.

### Western Blot Analysis

Total proteins were extracted and subjected to Western blot as described previously[8]. The polyclonal rabbit primary antibodies against MACC1 (Abcam, Cambridge, Massachusetts, USA), TWIST1 (Santa Cruz Biotechnology, Santa Cruz, CA, USA), VEGF-A antibodies (Proteintech, Chicago, IL, USA), and the secondary fluorescence goat anti-rabbit antibody (LI-COR, Lincoln, NE, USA) were used in this experiment. The blots were scanned using Odyssey imaging system (LI-COR).

### Quantitative Real-Time PCR

The sequence of the primer for TWIST1 is summarized in S4 Table. Total RNA was extracted using Trizol Kit (Invitrogen) and reverse transcribed by using the M-MLV RT kit (Promega, Madison, WI, USA). QRT-PCR was performed with SYBR Green dye (Roche Diagnostics, Mannheim, Germany).

### Statistical Analysis

All statistical analyses were performed using the SPSS 13.0 software (SPSS Inc., Chicago, IL, USA). The relationship between MVD and clinicopathologic variables was examined by the Chi-square test. DFS was defined as the interval from the date of operation to the date of the first recurrence. The DFS rate was estimated using the Kaplan-Meier method, and the survival differences were compared using the log-rank test. A multivariate analysis was performed using Cox’s regression model. The hazard ratios were presented with their 95% confidence intervals. The correlationship between MACC1 and MVD was determined by Spearman's rank test. Statistical comparison of different experimental groups in vivo and in vitro was performed by Student t-test. The value of P < 0.05 was considered statistical significant.

## Results

### Elevation of MVD and VEGF-A Are Related with Tumor Recurrence in GC Patients

**Fig 1:**
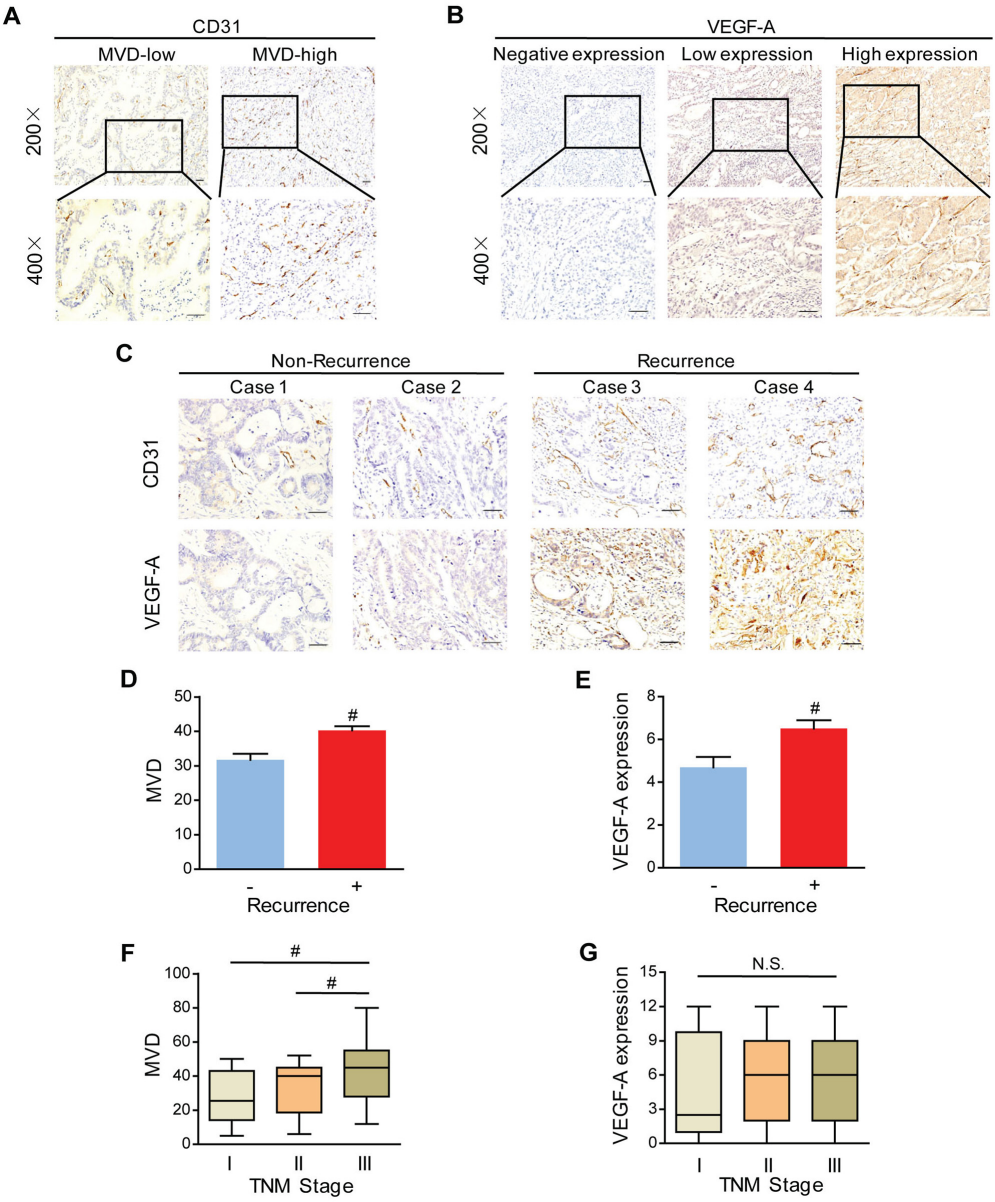
Microvessel density (MVD) and vascular endothelial growth factor A (VEGF-A) positively correlate with the recurrence. **(A)** Representative immunostaining of CD31 in GC tissues. **(B)** Representative pictures of VEGF-A stained in human GC tissues. **(C-E)** Representative photomicrographs **(C)** and Bar graph showing MVD **(D)** and VEGF-A expression **(E)** were higher in recurrence GC patients than in non-recurrence GC patients. Scale bar = 50 μm. ^#^P < 0.01, n = 65 and n = 94 for non-recurrence and recurrence patients, respectively. **(F&G)** In Stage I patients, MVD was lower **(F)** and average VEGF-A scores tended to be lower **(G)** than in Stage II and Stage III. ^#^P < 0.01 versus the corresponding control group, N.S., No significance; n = 26, n = 50 and n = 83 for Stage I, II, and III, respectively.

To assess the neovascularization index, MVD was determined by IHC staining of CD31 in 159 tissues of patients with Stage I-III GC. Representative examples of staining with high and low magnifications was indicated in Fig 1A. The median value of MVD, defined as cut-off value, was 36.8. We defined the values higher than cut-off as high MVD, otherwise as low MVD. Among the 159 GC patients, 106 (66.7%) were divided into MVD-high group and 53 (33.3%) were grouped into MVD-low group (Fig 1A). The association between MVD and the various clinicopathological parameters was listed in S5 Table. We found that MVD-high was more frequent in GC patients with recurrence (P = 0.001, Fig 1C and 1D). There was no significant association referring to gender, age, histological differentiation grade, depth of invasion (T stages), lymphatic metastasis (N stages), and overall TNM stages between two groups. However, our further analysis revealed that MVD was higher in Stage III than in both Stage II (P = 0.009) and Stage I (P = 0.007) (Fig 1F), while this was not the case considering individual T or N stage.

It has been documented that VEGF plays a pivotal role in stimulating tumor blood vessels formation and biologically correlates with MVD[20, 23]. VEGF-A was the most studied of VEGF family[20, 21]. Given the important role of VEGF-A in tumor angiogenesis, we conducted the immunostaining in 159 GC tissues (Fig 1B). According to VEGF-A staining scores, patients were divided into groups of negative (score 0–2), low (score 3–7) and high expressions (score 8–12). There were 108 VEGF-A positive patients, which took the majority (positives 67.9% vs. negatives 32.1%). Among them, 47 (29.6%) patients were defined as low expression, and 61 (38.4%) were as high expression. The relationship between VEGF-A expression and recurrence was further evaluated in GC. As a result, VEGF-A was found to be positively correlated with tumor recurrence of GC patients (P < 0.001, Fig 1C and 1E). Furthermore, though there was no statistically significant (P > 0.05), VEGF-A expression tended to be higher in Stage II-III than in Stage I judging from the box-plot (Fig 1G), which suggested a positive regulatory role of VEGF-A on MVD in GC progression.

### MACC1 Positively Correlates with MVD and VEGF-A in Human GC

**Fig 2:**
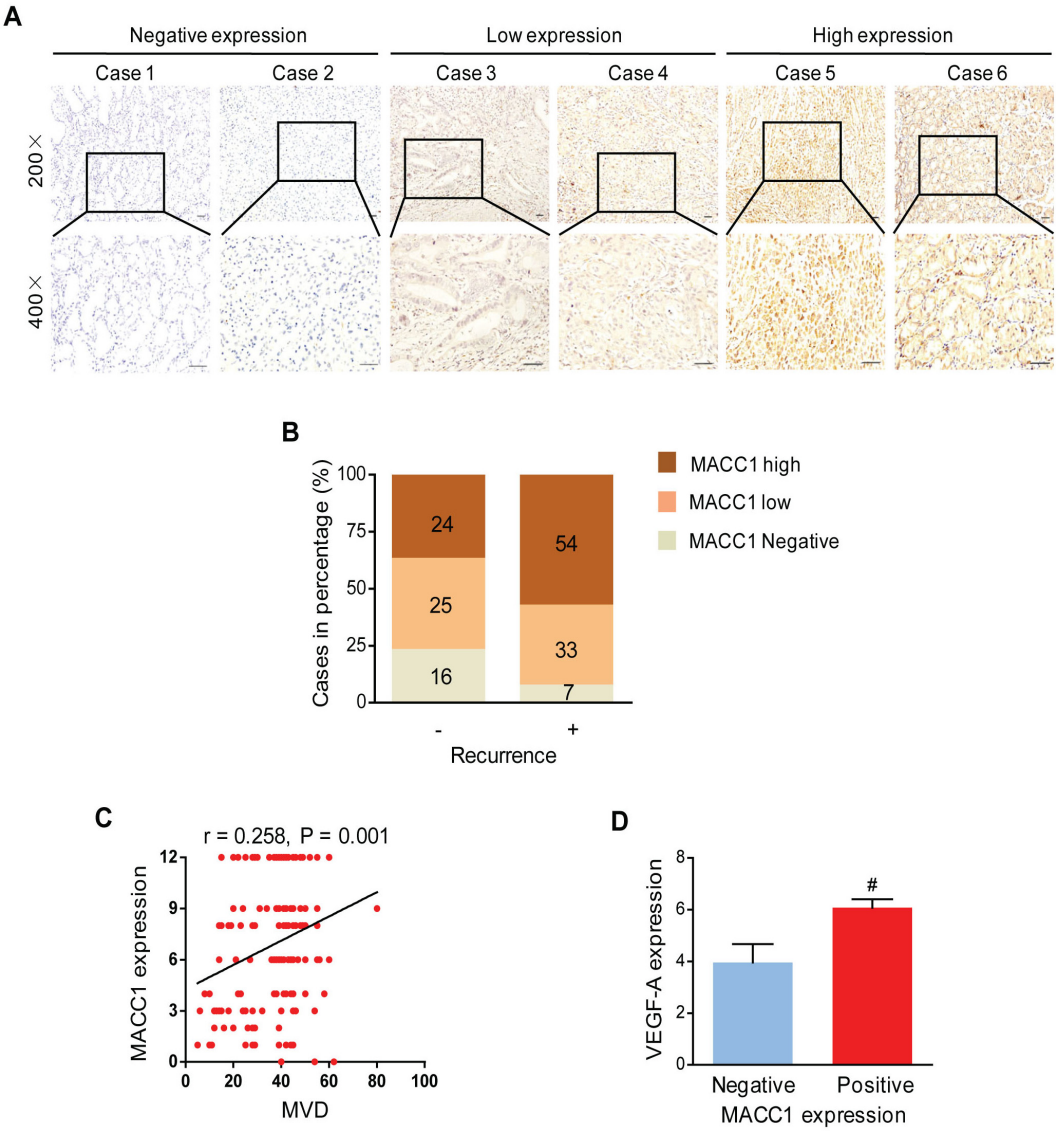
Expression of MACC1 is correlated with MVD, VEGF-A and recurrence. **(A)** Representative MACC1 stained images in GC tissues. Scale bar = 50 μm. **(B)** The frequency of negative, low, and high MACC1 expression in GC patients categorized by recurrence. **(C)** MACC1 expression scores were positively correlated with the MVD (r = 0.258, P = 0.001 and n = 159). **(D)** VEGF-A was higher in MACC1 positivity tumors. ^#^P < 0.01, n = 23 and n = 136 for MACC1-negative and MACC1-positive tumors, respectively. MACC1 positive expression included MACC1-low and -high expression.

To further clarify the role of MACC1 in the neovascularization of GC, we set out to determine whether MACC1 was correlated with MVD and angiogenic factor VEGF-A. Then, IHC staining was performed to detect MACC1 expression in GC tissue sections (Fig 2A). As indicated in Fig 2B, MACC1 showed higher expression in GC patients with recurrence than those without. Notably, MACC1 was positively correlated with MVD (r = 0.258, P = 0.001, Fig 2C) and increased VEGF-A expression level in GC tissues (P = 0.008, Fig 2D). Herein, these findings indicated that MACC1 correlated with MVD and VEGF-A in human GC.

### MACC1 and MVD Indicate Short DFS for GC Patients

**Fig 3:**
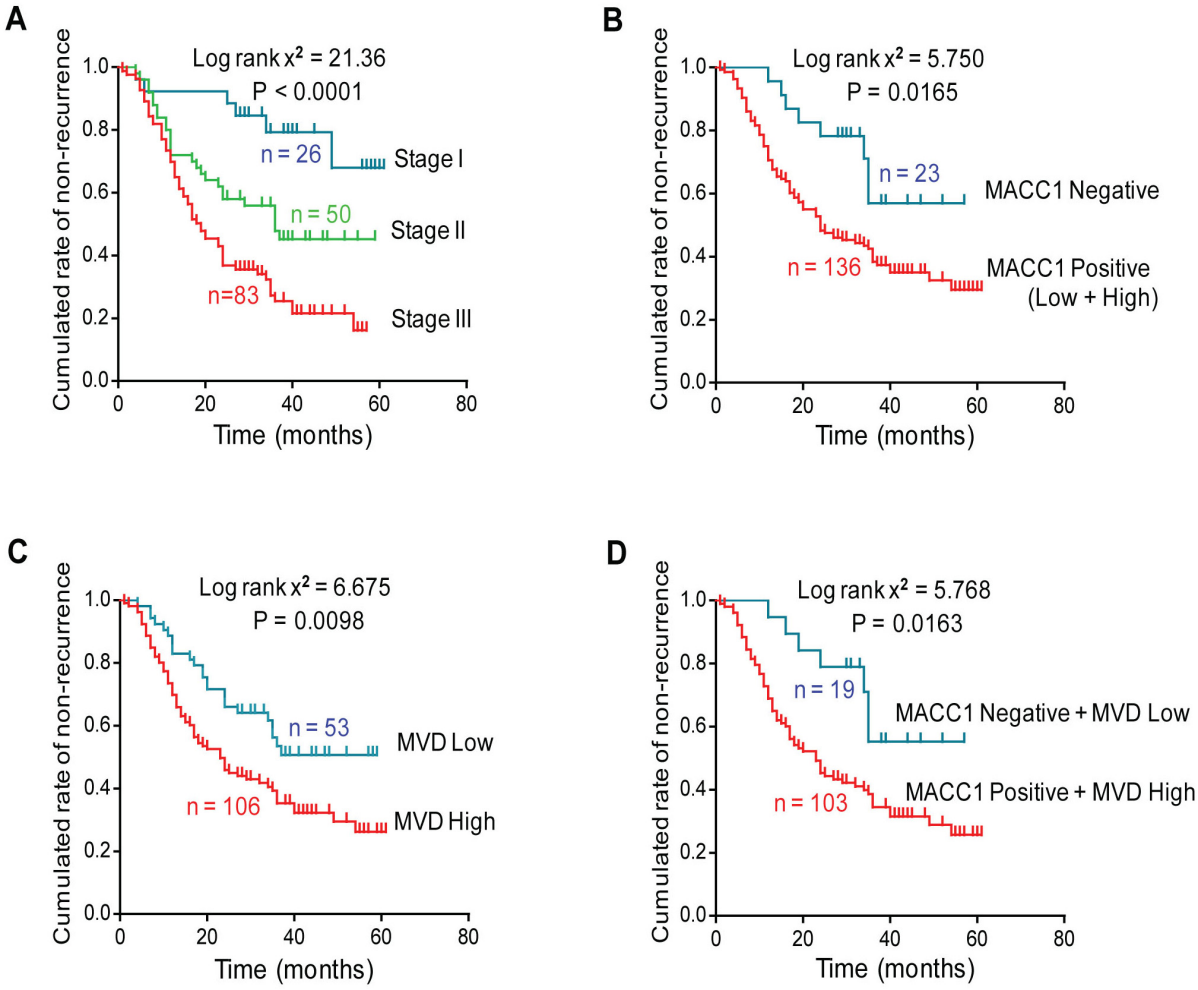
MACC1 and MVD indicate short disease-free survival (DFS) time in GC patients. **(A)** Kaplan-Meier analysis of GC patients DFS time in subgroups of Stage I-III. **(B-D)** Kaplan-Meier plot showing MACC1 positive expression and elevated MVD indicated short DFS of GC patients categorized by MACC1 expression **(B)**, MVD level **(C)** and by a combination of MACC1 positivity and MVD-high **(D)**.

According to the multivariate analysis using the Cox model, MVD (P = 0.043, HR = 1.737) and overall TNM stages (P < 0.001, HR = 2.559) were independent prognostic parameters predicting the risk of recurrence after radical resection in Stage I-III patients (S6 Table). Kaplan-Meier survival curve was plotted using aforementioned prognostic factors. Among the 159 patients, 94 (59.1%) relapsed and the median time to recurrence was 27.0 months. The DFS time was significantly shorter in patients with advanced TNM stage (P < 0.001, Fig 3A). Moreover, not only MACC1-positive or MVD-high alone affected DFS, the combination of both could also predict the short DFS in GC patients (Fig 3B–3D). Strikingly, the 3-year DFS rate for the combination of MACC1-positive and MVD-high patients was only 34.5%, whereas it was 55.3% for patients with tumors that were MVD-low and negative for MACC1 (Fig 3D).

### MACC1 Promotes GC Endothelium-Dependent Angiogenesis In Vivo and In Vitro

**Fig 4:**
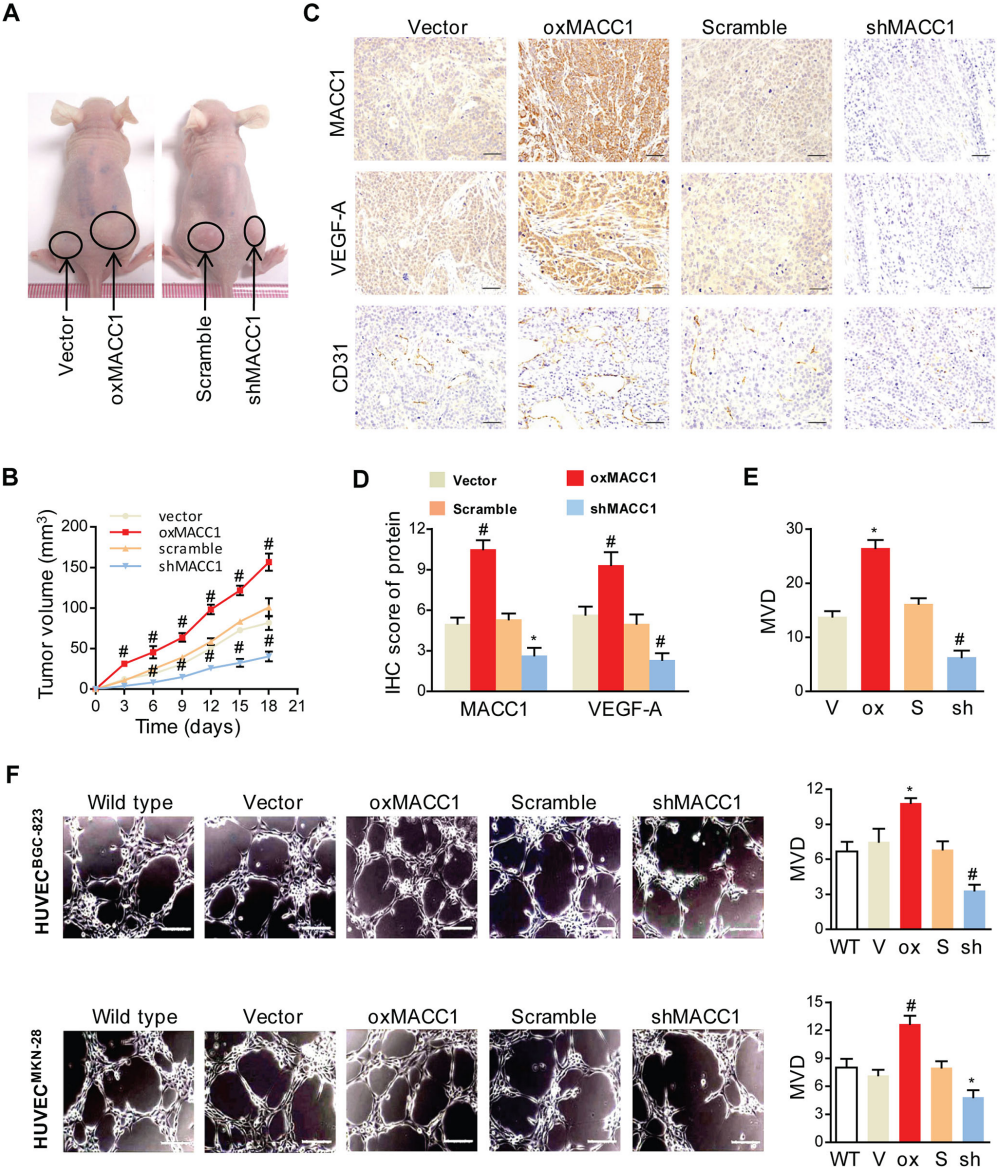
MACC1 promotes angiogenesis in vivo and in vitro. **(A)** Representative images of subcutaneous implanted overexpression of MACC1 (oxMACC1) and silencing of MACC1 (shMACC1) tumors of GC xenografts. **(B)** The tumor volume curves of GC xenografts at 18 days after inoculation. *P < 0.05; ^#^P < 0.01, n = 6. **(C-E)** Representative immunostaining of MACC1, VEGF-A, CD31 **(C)** and quantitation **(D&E)** from GC xenografts with oxMACC1, shMACC1 and their corresponding controls. Scale bar = 50 μm. *P < 0.05; ^#^P < 0.01, n = 6 vs. the corresponding control group. **(F)** Representative images of tube formation in three-dimensional culture (left panel) and quantitation (right panel) of HUVECs treated with conditioned media from BGC-823 or MKN-28 GC cells for 12 h. WT, wild type. V, vector. Ox, oxMACC1. S, scramble. Sh, shMACC1. Scale bar = 50 μm. *P < 0.05; ^#^P < 0.01, n = 3.

Angiogenesis is so important for tumor growth. Initially, we tested whether change of MACC1 expression could influence the growth of tumor in vivo. We successfully established BGC-823 cell lines with oxMACC1 and shMACC1 as described previously[8], then cells were injected subcutaneously into NOD-SCID nude mice (n = 6/group). As a result (Fig 4A and 4B), ectopic expression of MACC1 facilitated tumorigenesis in vivo: the average volume of the oxMACC1 tumors was markedly larger than that of the vector-controls at day 18, and shMACC1 injected mice displayed significantly minimal tumor volume. As shown in Fig 4C and 4D, the expression of VEGF-A was significantly increased in the oxMACC1 groups compared with the vector-control groups (P = 0.007). In contrast, VEGF-A score in the shMACC1 groups was significantly lower than in the scramble-control groups (P < 0.001). In both cell lines, the amount of MVD was found to be promoted by oxMACC1 and inhibited by shMACC1 (P = 0.025 and P = 0.002, Fig 4C and 4E).

In order to confirm the role of MACC1 in angiogenesis in vitro, we used a well-established three-dimensional (3D) culture model in indirect co-culture system. As shown in Fig 4F, the density of vascular tube-like structures was dramatically increased in HUVECs co-cultured with conditioned media from oxMACC1 BGC-823 and MKN28 cells (P = 0.05 and P = 0.003), while that was significantly decreased from shMACC1 BGC-823 and MKN28 cells, compared with the corresponding vector- and scramble-control cells at 12 h (P = 0.006 and P = 0.02). Taken together, these results revealed that MACC1 accelerated tumor growth and induced endothelium-dependent angiogenesis of GC.

### MACC1 Promotes Angiogenesis by Upregulating TWIST1 Expression

**Fig 5:**
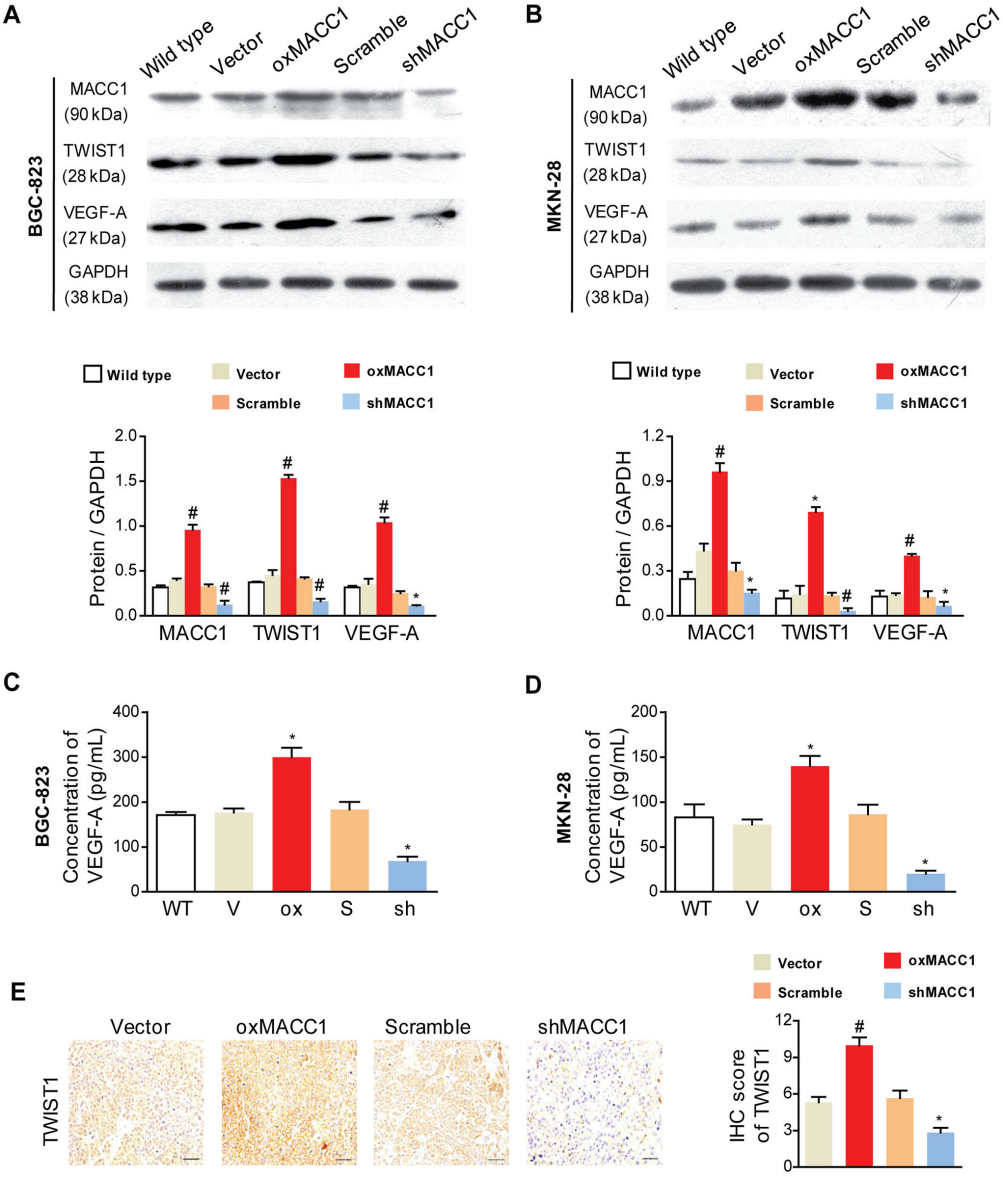
MACC1 upregulates TWIST1 and VEGF-A expression in GC cells. **(A&B)** Western blot analysis (upper panel) and quantitation (lower panel) of MACC1, TWIST1, and VEGF-A expression in response to overexpression or silencing of MACC1 (oxMACC1 and shMACC1) in BGC-823 and MKN-28 GC cells. *P < 0.05; ^#^P < 0.01, n = 3. WT, wild type. GAPDH was used as a loading control. **(C&D)** ELISA analysis of VEGF-A secreted protein levels in the supernatants of oxMACC1 or shMACC1 GC cells. **(E)** Representative images (left panel) and quantitation (right panel) of TWIST1 immunostaining in xenograft GC tissues. WT, wild type. V, vector. Ox, oxMACC1. S, scramble. Sh, shMACC1. Scale bar = 50 μm. *P < 0.05; ^#^P < 0.01, n = 3.

We previously reported that TWIST1 was an essential factor in MACC1-induced VM of GC cells. MACC1 could transcriptionally upregulate TWIST1[4]. This prompted us to investigate whether TWIST1 engaged in the MACC1-induced endothelium-dependent angiogenesis of GC. The results showed in Fig 5A and 5B suggested that the expression of TWIST1 was obviously upregulated in oxMACC1 GC cell lines (P < 0.001 and P = 0.024), but was significantly downregulated in shMACC1 GC cell lines compared with the corresponding control cells (P = 0.002 and P < 0.001). Similar conclusions have been reached in the ELISA assay (S2 Fig). Next, we detected the protein expression of VEGF-A in two GC cell lines (Fig 5A and 5B), VEGF-A was greatly increased by MACC1 upregulation (P < 0.001 and P = 0.008), but was decreased by shMACC1 (P = 0.039 and P = 0.041). Similar results were obtained by the ELISA assay (Fig 5C and 5D). In addition, IHC staining in subcutaneous tumors with GC xenografts showed that TWIST1 and VEGF-A were significantly greater for oxMACC1 cells versus vector-controls, and was markedly decreased for shMACC1 cells versus scramble-controls (P = 0.003 and P = 0.026, Figs 4C and 5E).

Up till now, it seems that TWIST1 is involved in MACC1-induced endothelium-dependent angiogenesis, so there comes another possibility: is TWIST1 necessary in angiogenesis induced by MACC1?

### TWIST1 Is Necessary for MACC1-Induced Angiogenesis in GC Cells

To determine whether TWIST1 is important for MACC1-induced angiogenesis in GC, we overexpressed the ectopic TWIST1 gene (oxTWIST1) in the shMACC1 cells and knocked down the endogenous TWIST1 (siTWIST1) in the oxMACC1 cells. Three independent siRNA constructs against Twist1 in ORF region were used (S2 Table). In mRNA levels, all of the three siTWIST1 successfully suppressed TWIST1 expression and the most efficient one was used to transfect GC cells (S3 Fig). The efficiency of overexpressing or silencing was verified by qRT-PCR and Western blot.

**Fig 6:**
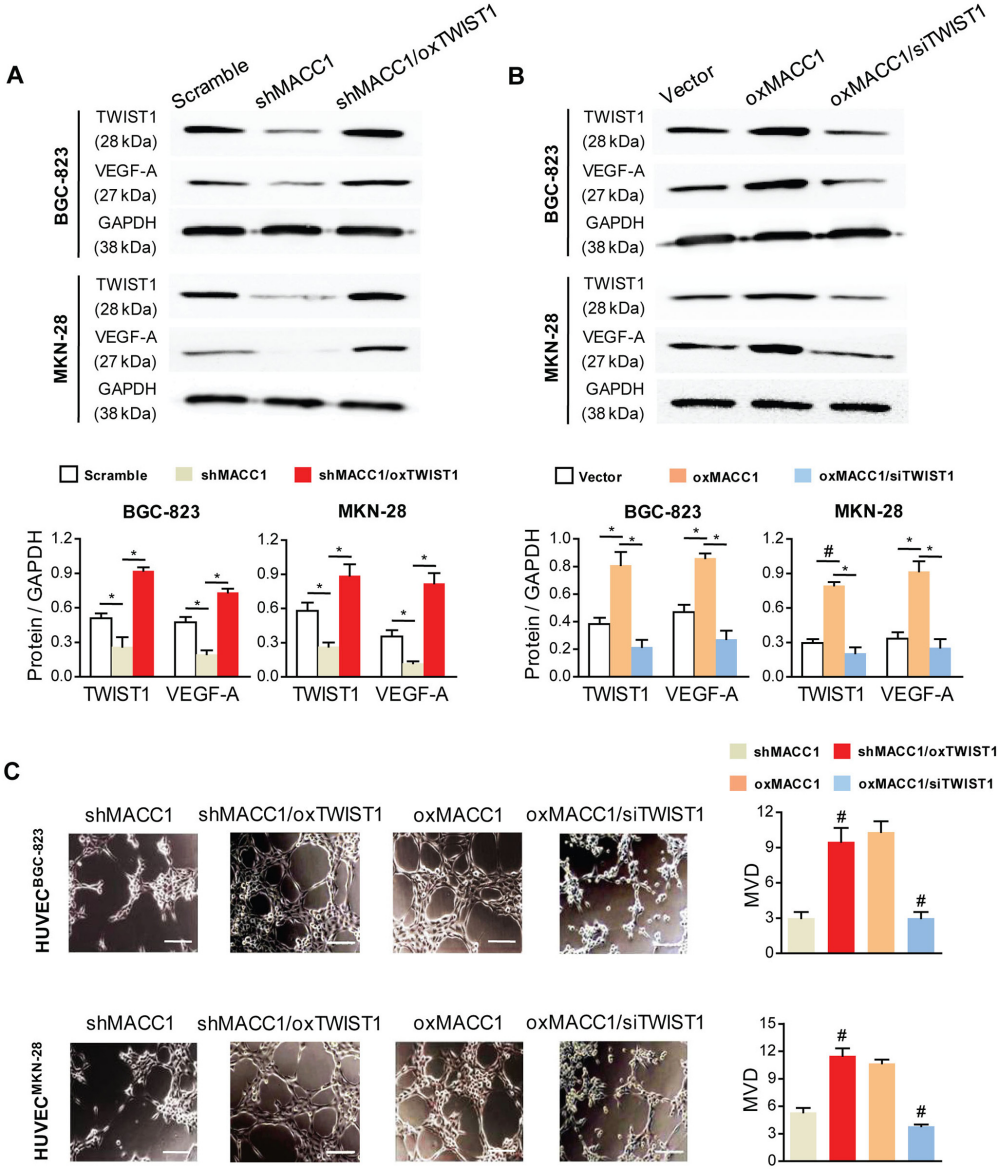
TWIST1 is required for MACC1-induced angiogenesis in BGC-823 and MKN-28 cells. **(A&B)** Western blot analysis (upper panel) and quantitation (lower panel) of VEGF-A expression in silencing of MACC1 (shMACC1) GC cells that overexpressed TWIST1 (oxTWIST1) and overexpression of MACC1 (oxMACC1) GC cells that silenced TWIST1 (siTWIST1). *P < 0.05; ^#^P < 0.01, n = 3. **(C)** Representative micrographs of vascular tube-like formation in three-dimensional cultures of HUVECs treated with the supernatants of shMACC1/oxTWIST1 or oxMACC1/siTWIST1, and with corresponding controls at 12 h. Scale bar = 50 μm. *P < 0.05; ^#^P < 0.01, n = 3 in each group.

Next, we detected the protein expression level of VEGF-A by Western blot in GC cell lines. In both cell lines, changes of VEGF-A protein expression was obviously increased by oxTWIST1 (P = 0.015 and P = 0.02, Fig 6A), but that was significantly reduced by siTWIST1 (P = 0.022 and P = 0.01, Fig 6B). In the tube formation assay, the density of tube formation was promoted by oxTWIST1 (P = 0.007 and P < 0.001) and was inhibited by siTWIST1 (P < 0.001 and P < 0.001, Fig 6C). Collectively, the results from these complementary sets of overexpression and knockdown experiments demonstrated that TWIST1 was necessary and sufficient to participate in MACC1-induced endothelium-dependent angiogenesis, meaning that MACC1 induced angiogenesis via activation of the TWIST1/VEGF-A angiogenic axis in GC.

## Discussion

Angiogenesis is thought to be a crucial step in cancer progression, and multiple studies have demonstrated its roles in aggressive malignancies[24]. Despite that previous studies have found multiple effective regulating factors promoting endothelium-dependent angiogenesis[14, 15, 18, 25], the potential role of MACC1 in GC angiogenesis remains unknown. In this study, we mainly investigated the role of MACC1 on the endothelium-dependent angiogenesis of GC. The results showed that upregulation of MACC1 had significant correlation with higher MVD and VEGF-A expression in GC. The combination of MACC1 positivity and high MVD predicted short DFS in GC patients. Furthermore, we found that MACC1 increased the expression of TWIST1 in GC cell lines and facilitated tube formation of HUVECs. MACC1 not only elevated the VEGF-A level in vitro, but also accelerated the tumor growth and induced the microvessel formation in vivo. Therefore, the present data from our studies indicate that MACC1 is an oncogenic enhancer for endothelium-dependent angiogenesis and tumor progression in GC.

We previously reported that MACC1 was impaired by metabolic stress via AMP-activated protein kinase (AMPK) activation, and involved in tumor energy metabolism by enhancing the Warburg effect[9]. MACC1 promoted GC cell proliferation and invasion by inducing the epithelial-mesenchymal transition (EMT) through activation of HGF/ c-Met signaling pathway[8], and induced VM formation of GC by HGF/c-Met-TWIST1/2-VE-cadherin/VEGFR2 signaling axis[4]. In addition, it has been reported that MACC1 participates in various signaling networks, including Akt/β-catenin[26] and Ras/Erk[27], the activation of which was also reported to enhance TWIST1 expression[28–30]. In pancreatic cancer cell lines, HGF/c-Met has been showed to transmit intracellular signal via MAPK pathway[31], and is essential to phosphorylate and stabilize TWIST1 in breast cancer[28]. Our previous study provided the first evidence that MACC1 transcriptionally upregulated TWIST1. Meanwhile, we demonstrated that HGF facilitated the nuclear translocation of MACC1 and upregulation of TWIST1 to promote VM in GC, whereas a c-Met inhibitor antagonized this process[4]. However, MACC1 as the key regulating factor for the HGF/c-Met/MAPK signaling pathway[5], its relationship with TWIST1 in the endothelium-dependent angiogenesis of GC has not yet been found. In the present study, our results proved that MACC1 upregulated TWIST1 expression and induced the vascular tube-like formation in HUVECs. Therefore, MACC1 as a transcriptional factor could transcriptionally modulate endothelium-dependent angiogenesis and mediate TWIST1 upregulation, which is responsible for the angiogenesis and tumor progression of GC.

To date, the predominant role of TWIST1 in tumor progression is thought to be inducing EMT[32]. Very few studies revealed that TWIST1 promoted endothelium-dependent angiogenesis in breast cancer[33] and in hepatocellular carcinoma[34] by recruiting macrophages, altering metalloproteinase 9 expression, and activating of VEGF-A signaling[19]. To further investigate whether TWIST1 is also as a critical mediator in MACC1-induced endothelium-dependent angiogenesis in GC, we identified that the expression of TWIST1 was significantly increased in the presence of MACC1. Moreover, silencing TWIST1 greatly suppressed MACC1-mediated tube formation of HUVECs, suggesting that TWIST1 is required for MACC1-mediated angiogenesis of GC.

The mechanisms underlying the VEGF-A-induced angiogenesis of cancer cells in both basic and clinical research studies have been intensively investigated[35–37]. It has been suggested that VEGF-A acts as a switch to promote angiogenesis[38]. Consistent with the critical role of VEGF-A in angiogenesis, the present study validated that the expression of VEGF-A and MVD, as well as tumor growth were significantly increased in MACC1 overexpression GC cells. Overall, these results indicate that VEGF-A signaling pathway is involved in MACC1/TWIST1-induced endothelium-dependent angiogenesis of GC.

## Conclusions

**Fig 7:**
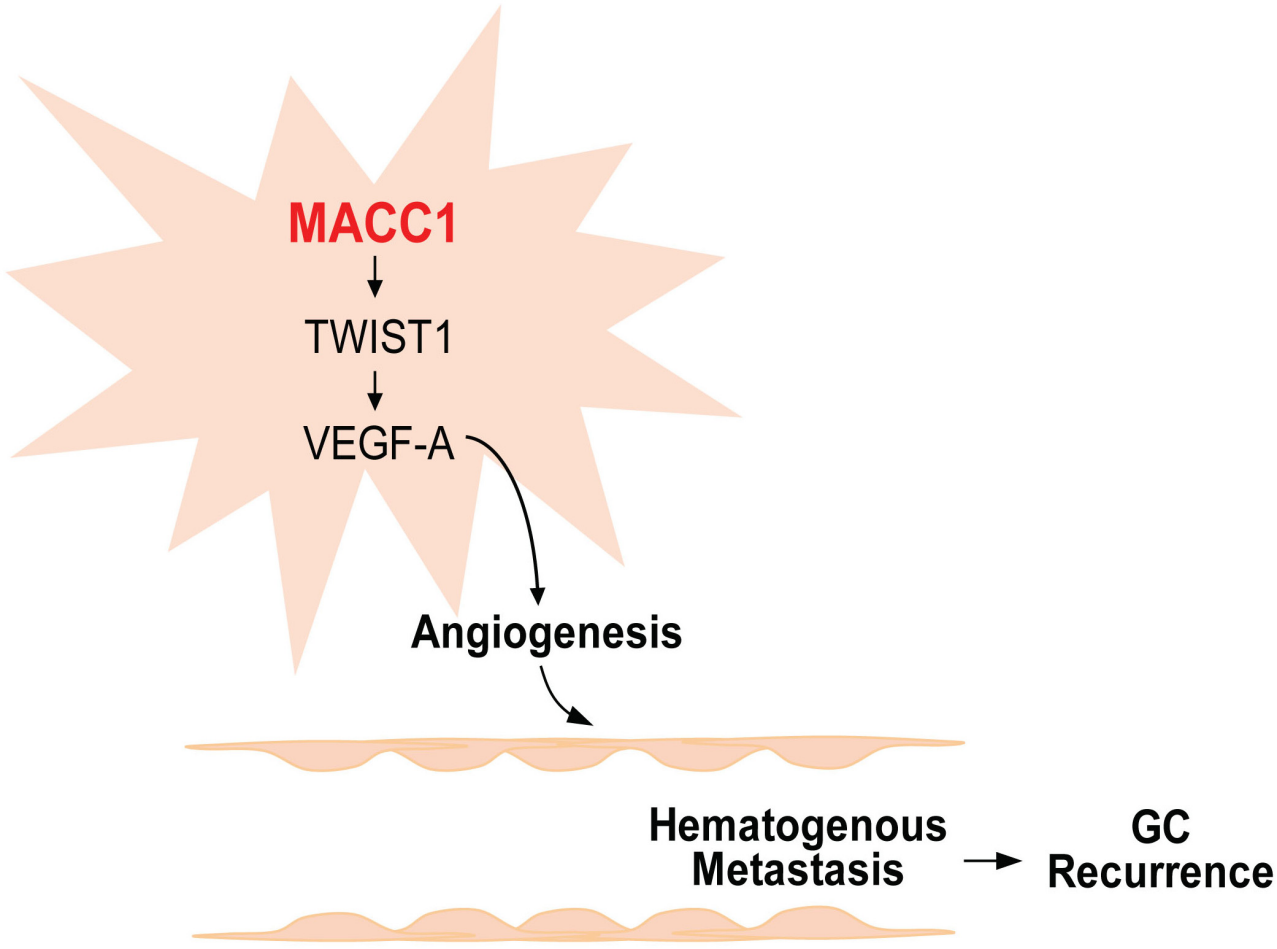
Summarizing diagram of the MACC1-dependent signaling axis in endothelium-dependent angiogenesis. Summarizing diagram of this study, MACC1 overexpression in gastric cancer (GC) activates the TWIST1 angiogenetic axis, which leads to upregulation of VEGF-A, and facilitates angiogenesis. Then angiogenesis promotes GC cell growth. Hence, higher rates of postoperative recurrence are seen in GC patients with increased expression of MACC1 and elevated MVD.

In conclusion, our study provided both clinical and experimental evidence for the function of MACC1 in endothelium-dependent angiogenesis of GC. Ectopic expression of MACC1 promoted tumor proliferation, significantly upregulated TWIST1 expression in vivo, and induced vascular tube-like formation in vitro. These findings indicate that MACC1 promotes endothelium-dependent angiogenesis in GC by the signaling pathway displayed in Fig 7. Understanding how MACC1 is involved in GC pathogenesis will be useful for developing potential therapeutic targets in the management of GC.

## Supporting Information

S1 Fig In mRNA level, the expression of MACC1 was positively correlated with VEGF-A in GC tissues (TCGA; n = 387, r = 0.224, P < 0.001). (TIF)

S2 Fig ELISA analysis of TWIST1 protein levels in the supernatants of overexpression or silencing of MACC1 (oxMACC1 and shMACC1) GC cells. WT, wild type. V, vector. Ox, oxMACC1. S, scramble. Sh, shMACC1. *P < 0.05; ^#^P < 0.01, n = 3.
(TIF)

S3 Fig The efficiency of three silencing TWIST1 (siTWIST1) in ORF region was verified by quantitative real-time PCR (qRT-PCR). *P < 0.05; ^#^P < 0.01, n = 3 vs. the corresponding control group.
(TIF)

S1 Table Primer sequences for TWIST1 overexpressing. (TIF)

S2 Table Three interference sequences for TWIST1 silencing (ORF region). (TIF)

S3 Table List of proteins tested by antibodies and features of the corresponding antibodies. (TIF)

S4 Table Primer sequences for quantitative real-time PCR. (TIF)

S5 Table Correlation between MVD and clinicopathologic characteristics in 159 Stage I-III GC patient specimens. (TIF)

S6 Table Multivariate analysis of various prognosis parameters in 159 GC patients using Cox regression model. (TIF)
