# Supplementary figures and images for: MACC-1 Promotes Endothelium-Dependent Angiogenesis in Gastric Cancer by Activating TWIST1/VEGF-A Signal Pathway
Source: PLoS One. 2016 Jun 9;11(6):e0157137. doi: 10.1371/journal.pone.0157137 (PMC4900635; doi:10.1371/journal.pone.0157137)

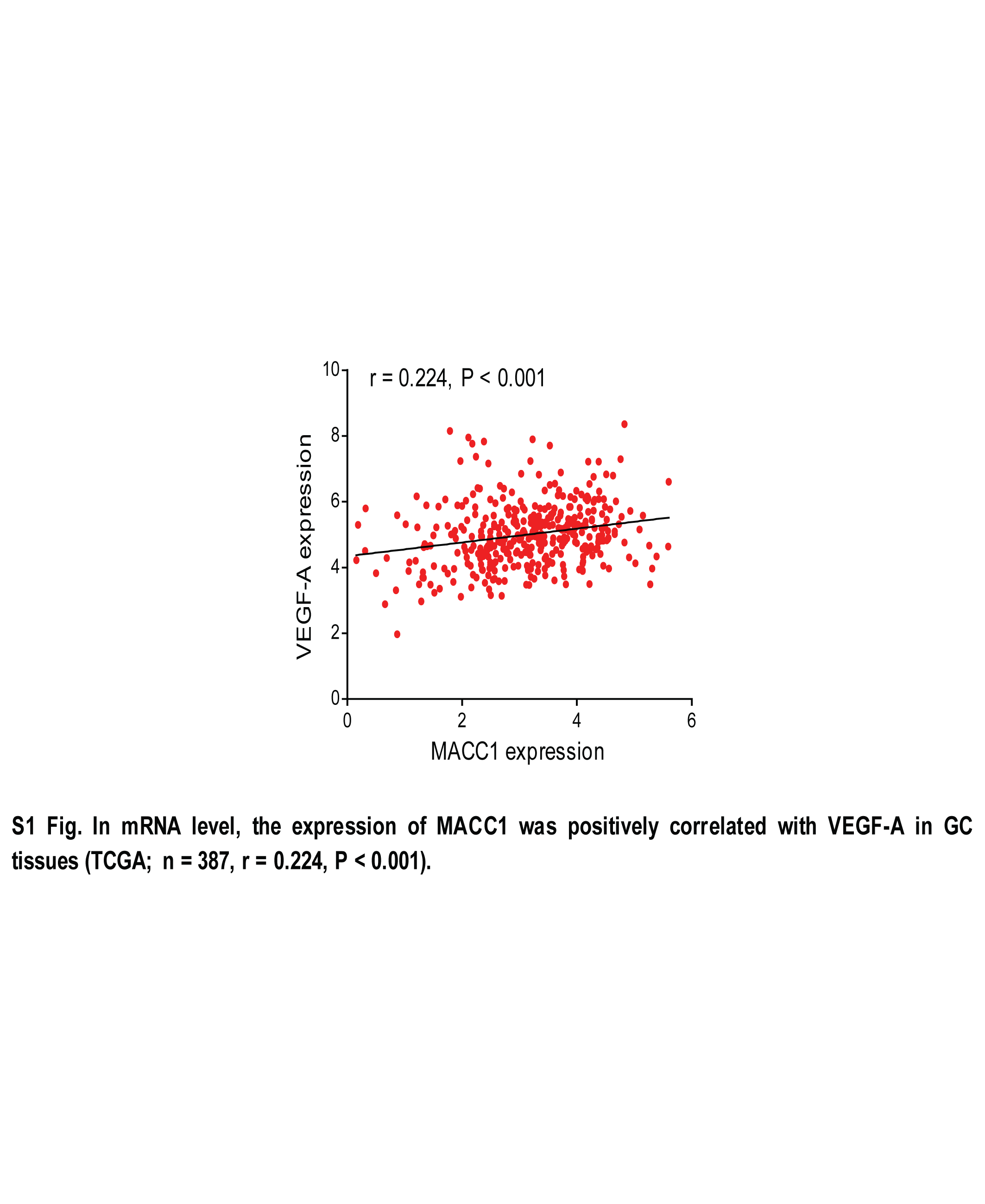

Supplement: S1 Fig — (TIF) [file pone.0157137.s001.tif]

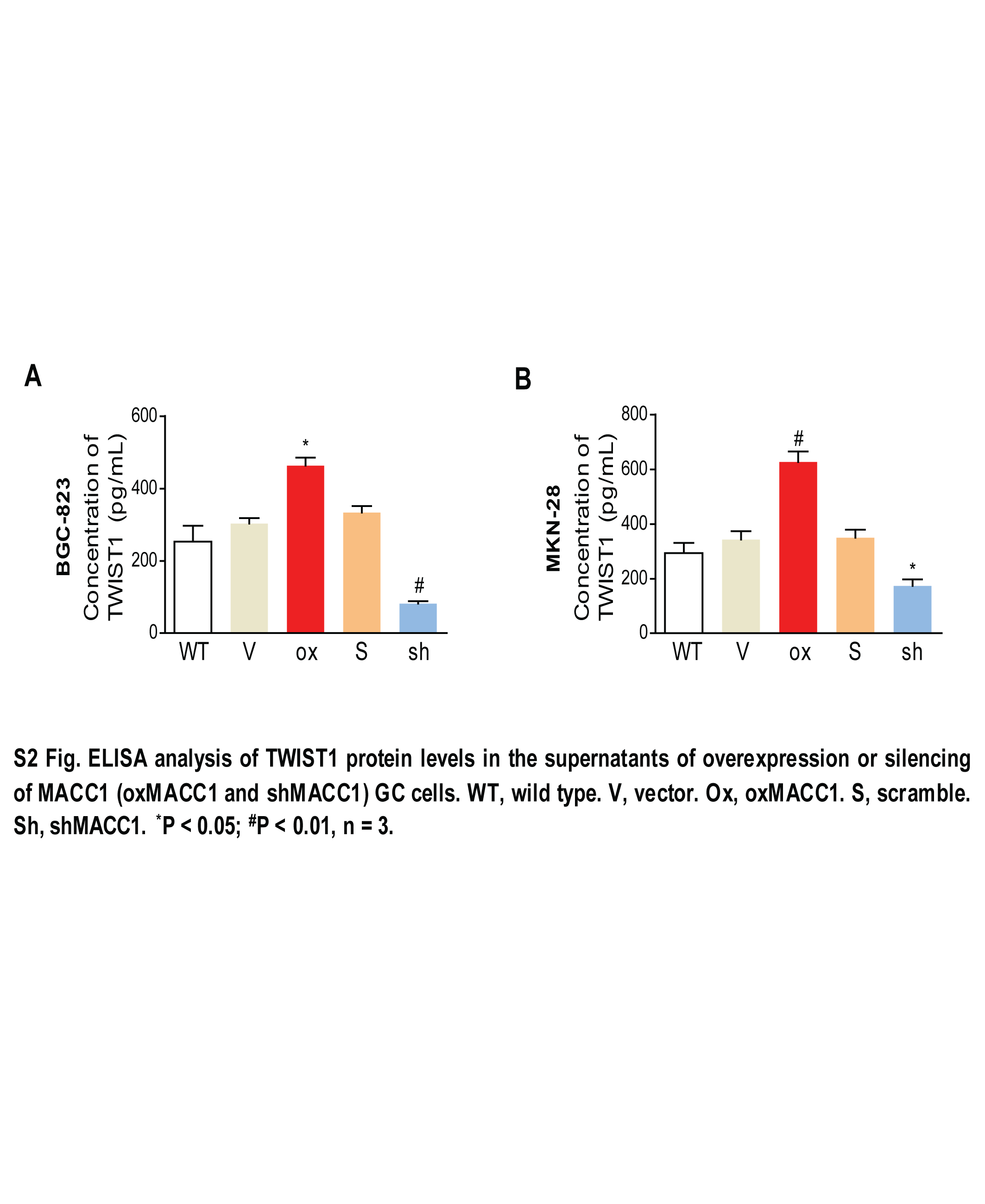

Supplement: S2 Fig — WT, wild type. V, vector. Ox, oxMACC1. S, scramble. Sh, shMACC1. *P < 0.05; #P < 0.01, n = 3. (TIF) [file pone.0157137.s002.tif]

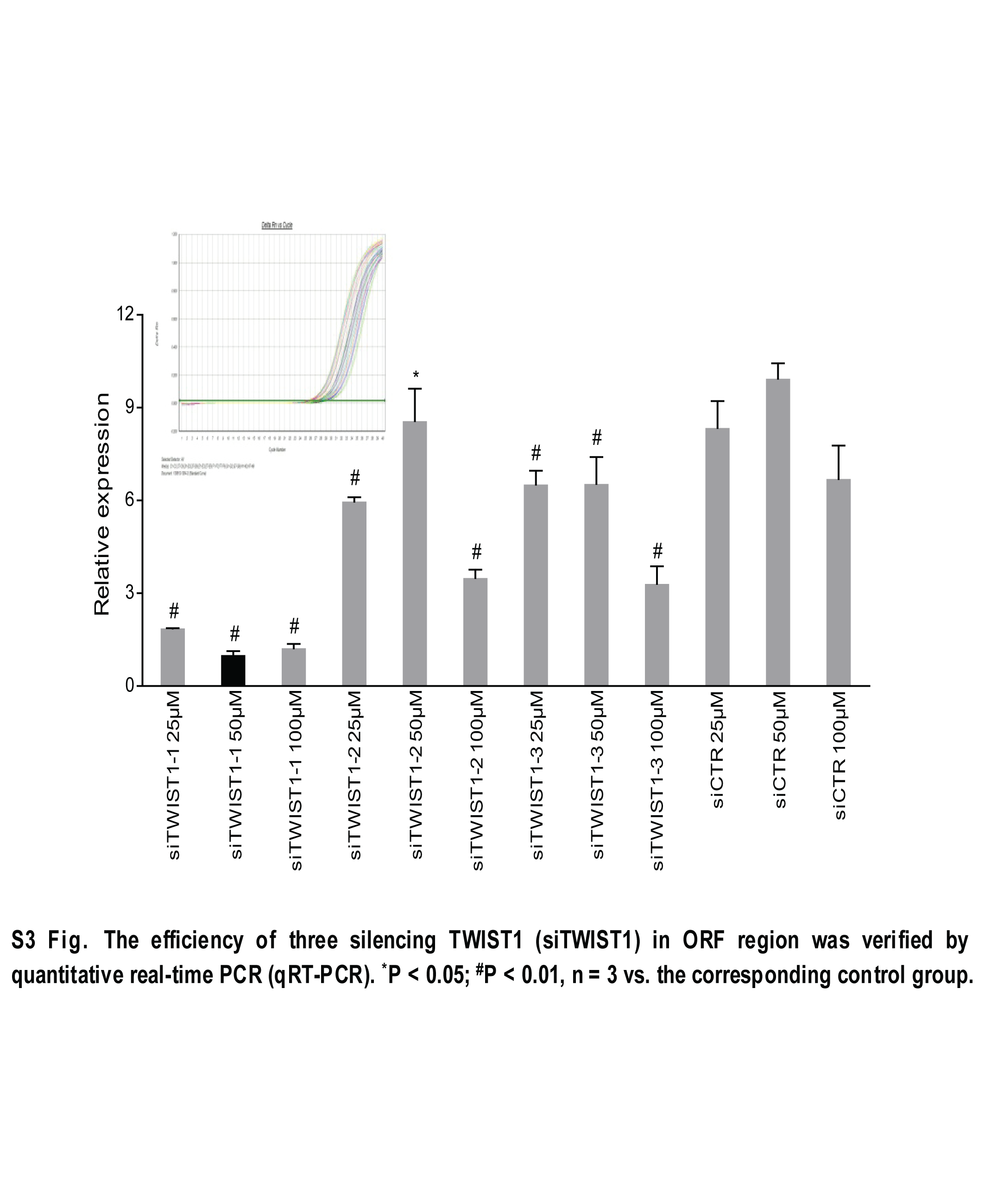

Supplement: S3 Fig — *P < 0.05; #P < 0.01, n = 3 vs. the corresponding control group. (TIF) [file pone.0157137.s003.tif]

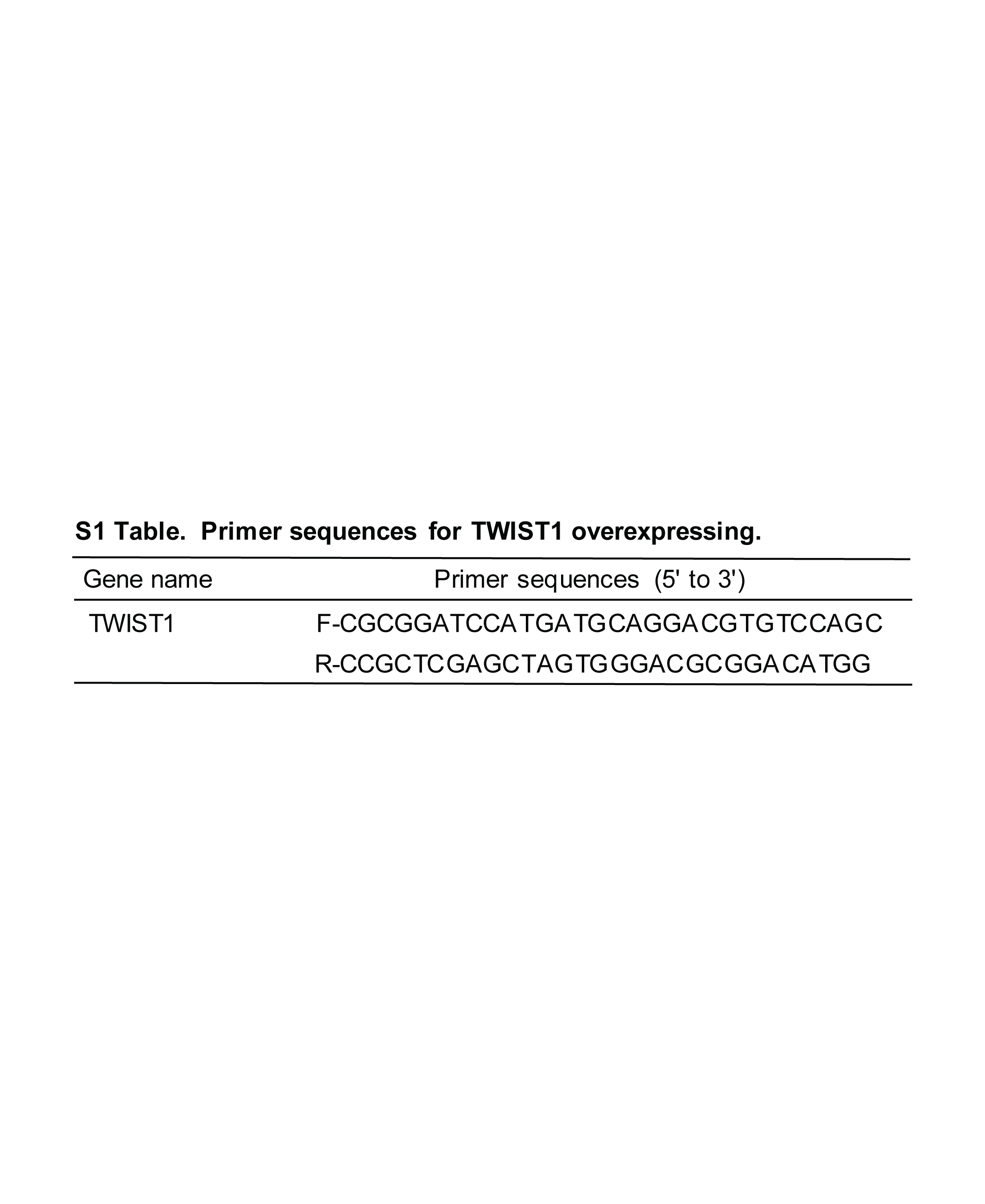

Supplement: S1 Table — (TIF) [file pone.0157137.s004.tif]

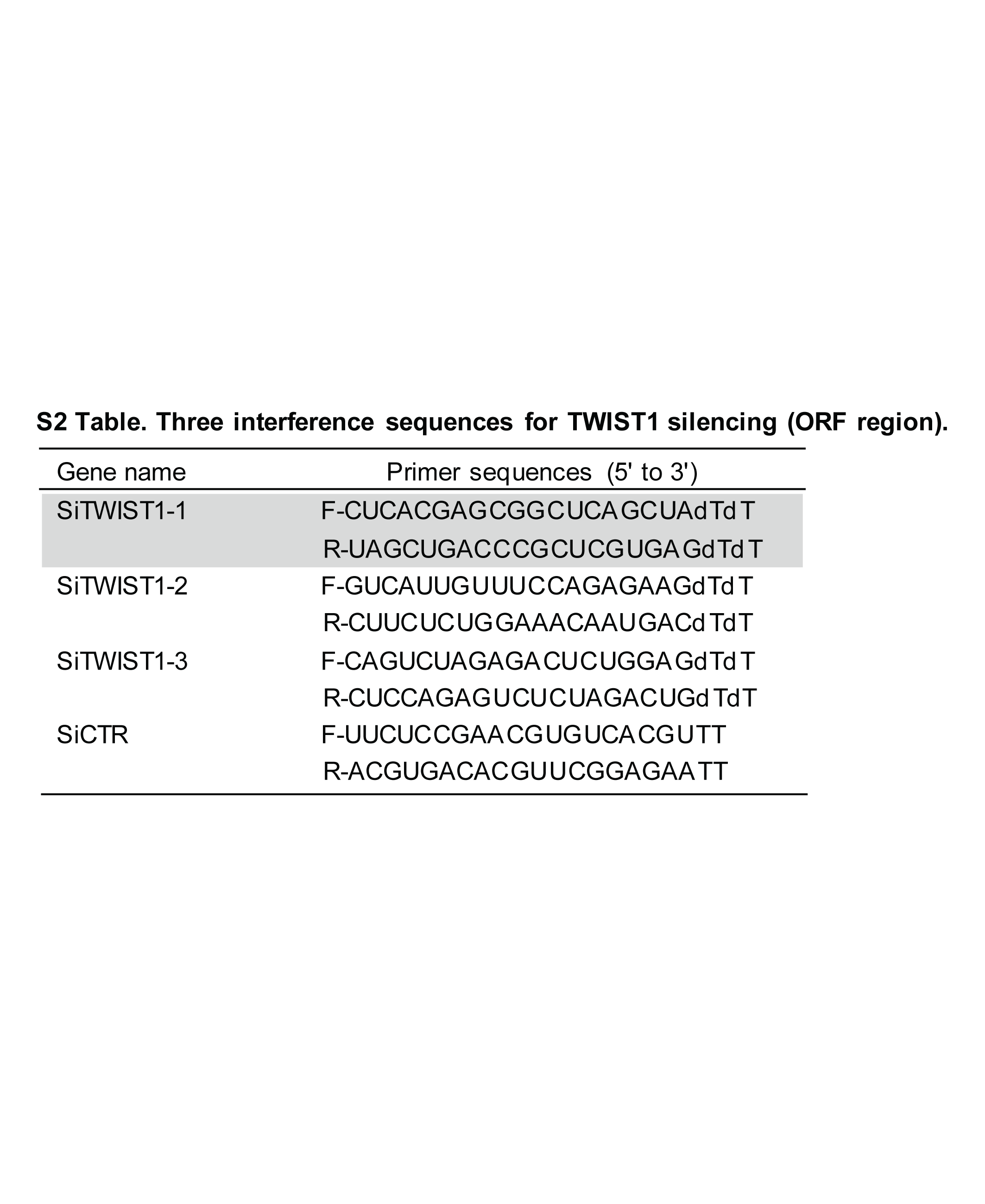

Supplement: S2 Table — (TIF) [file pone.0157137.s005.tif]

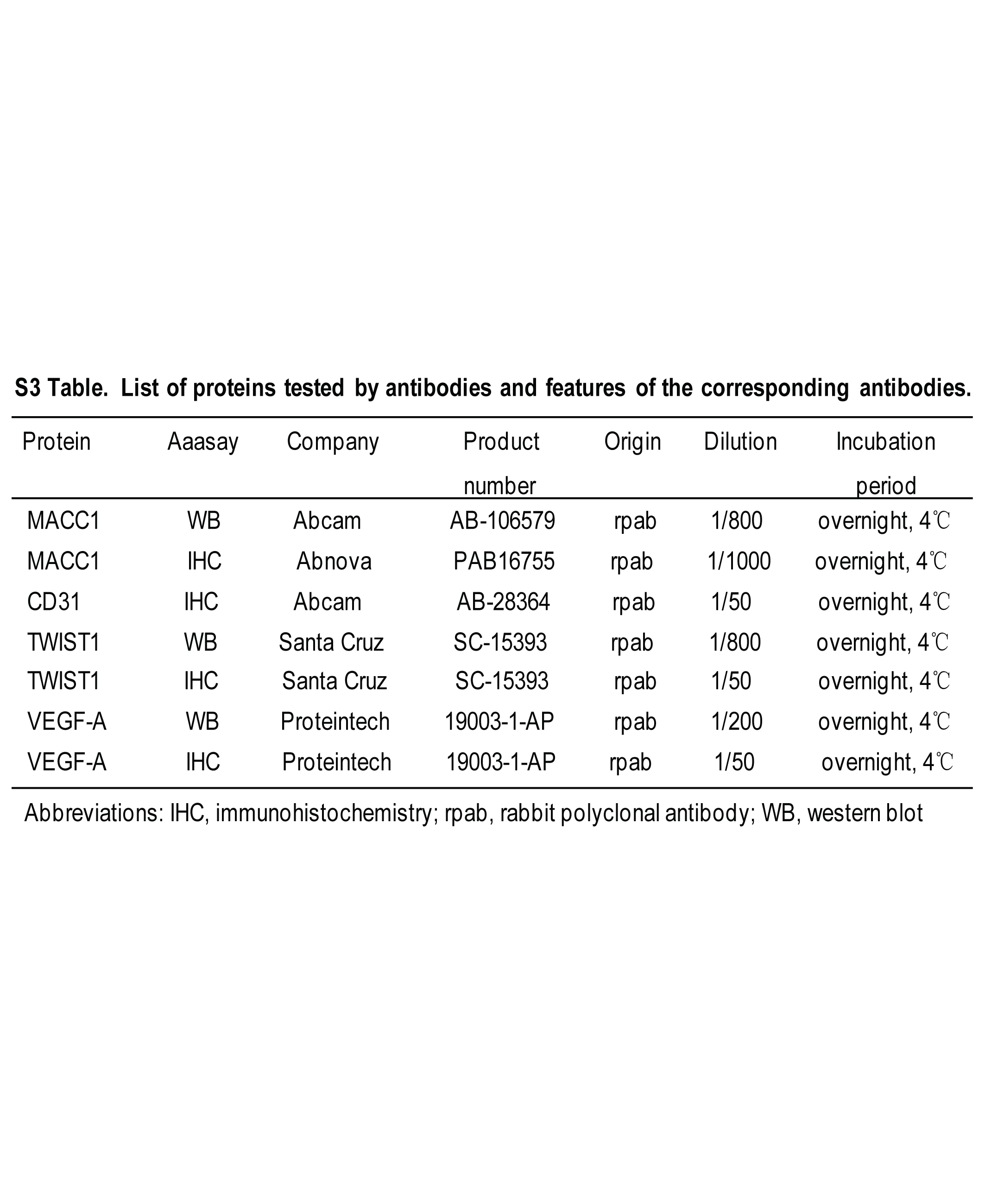

Supplement: S3 Table — (TIF) [file pone.0157137.s006.tif]

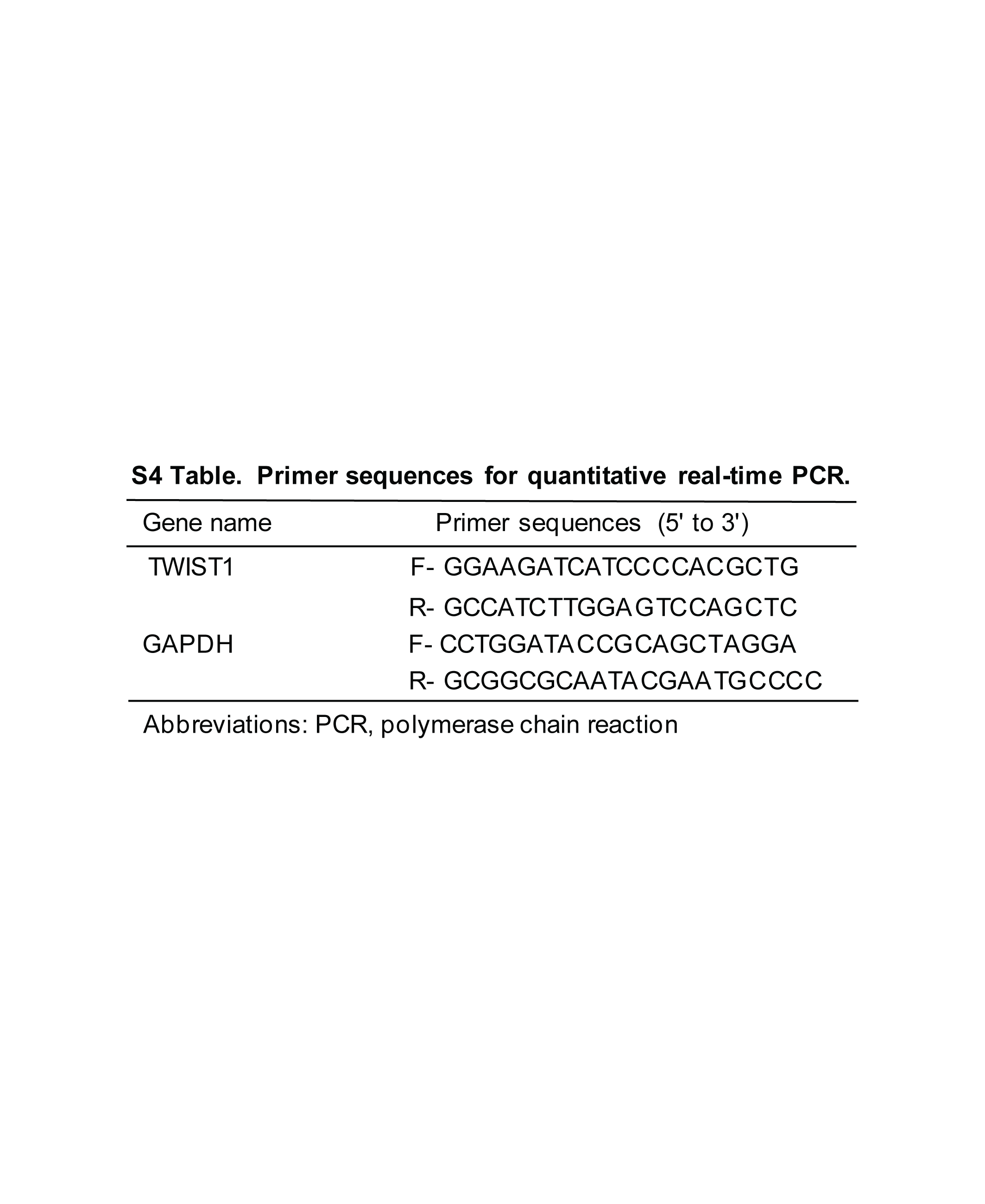

Supplement: S4 Table — (TIF) [file pone.0157137.s007.tif]

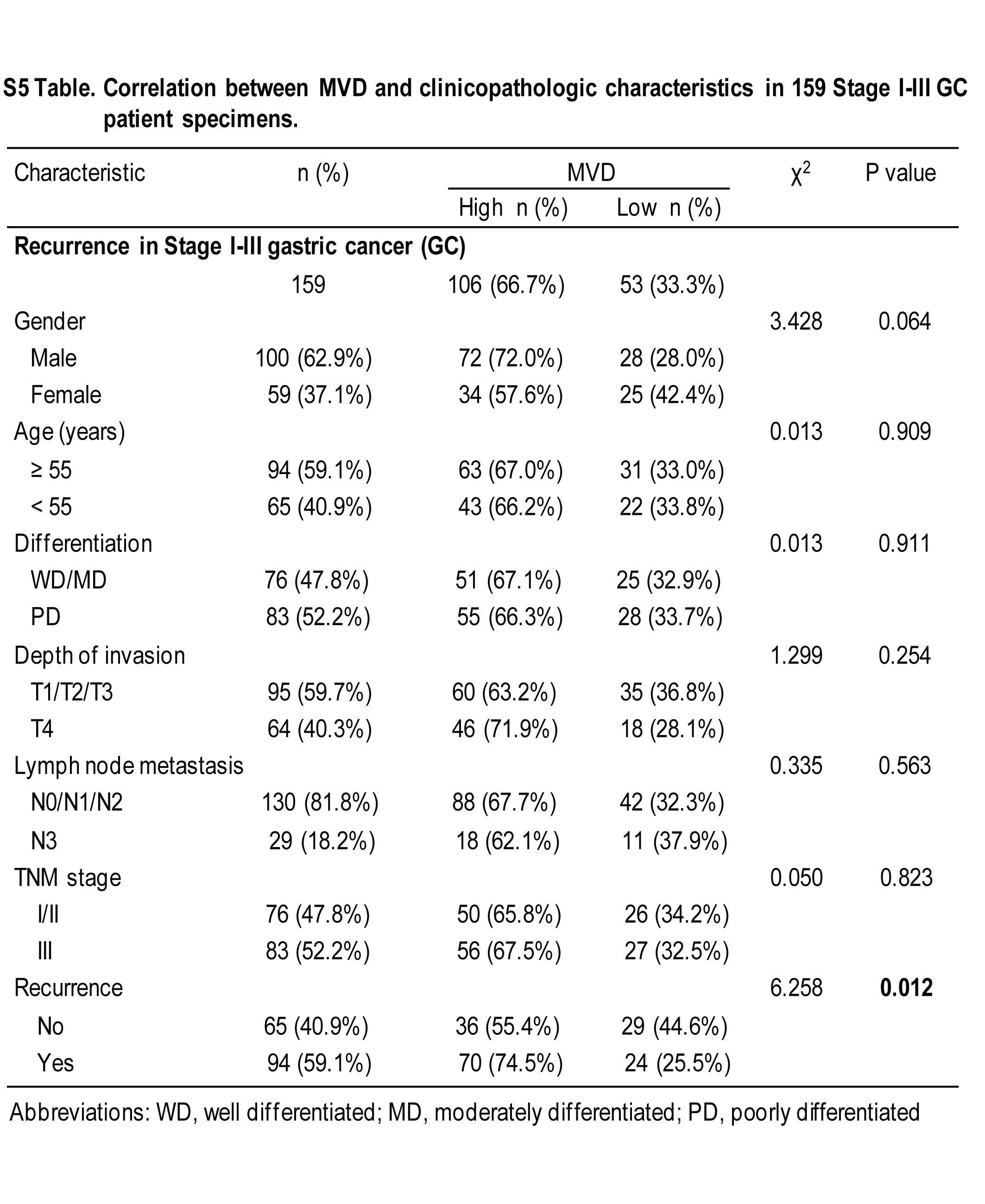

Supplement: S5 Table — (TIF) [file pone.0157137.s008.tif]

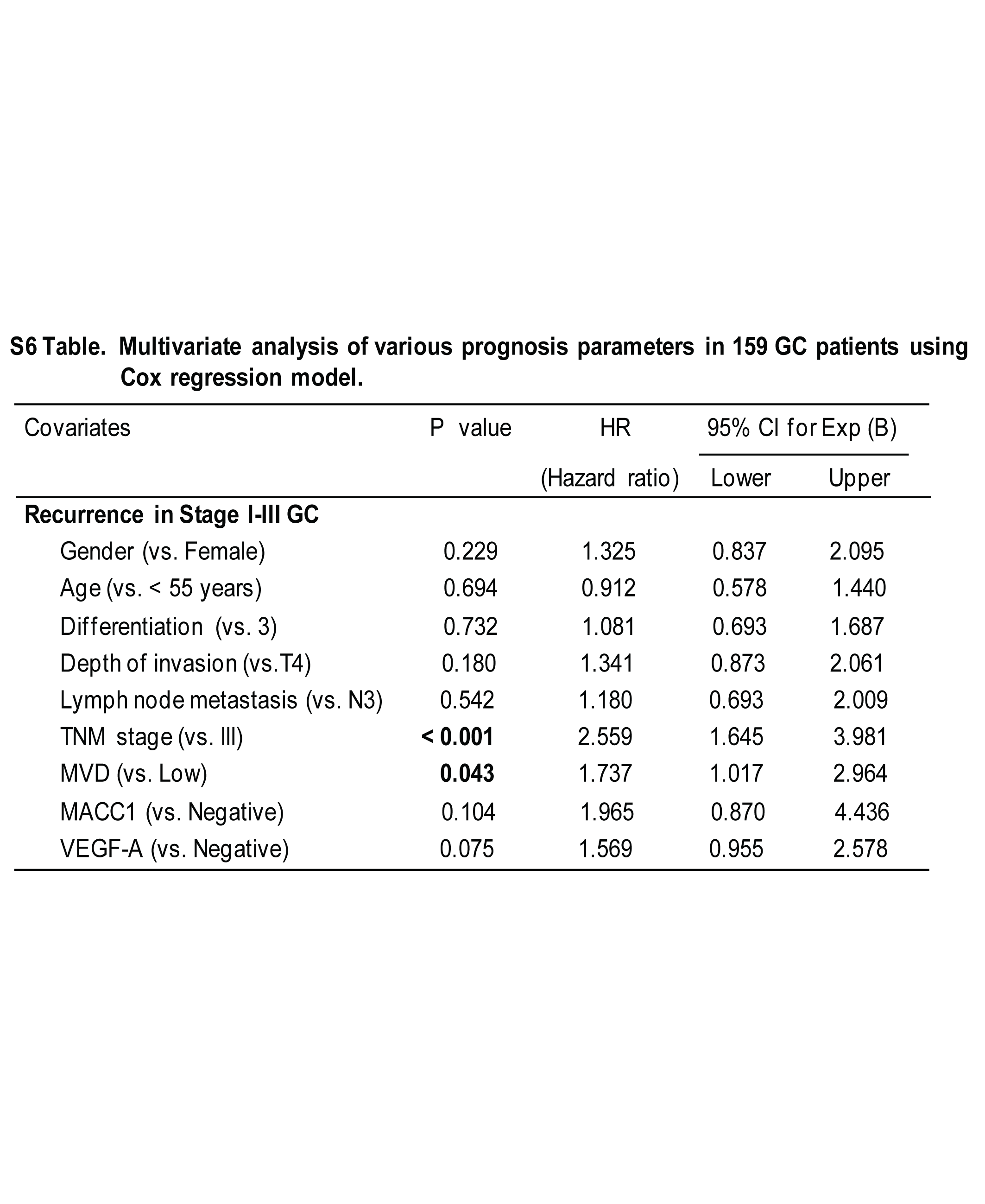

Supplement: S6 Table — (TIF) [file pone.0157137.s009.tif]
